# IP-10 Promotes Latent HIV Infection in Resting Memory CD4^+^ T Cells *via* LIMK-Cofilin Pathway

**DOI:** 10.3389/fimmu.2021.656663

**Published:** 2021-08-10

**Authors:** Zhuo Wang, Xiaowan Yin, Meichen Ma, Hongchi Ge, Bin Lang, Hong Sun, Sijia He, Yajing Fu, Yu Sun, Xiaowen Yu, Zining Zhang, Hualu Cui, Xiaoxu Han, Junjie Xu, Haibo Ding, Zhenxing Chu, Hong Shang, Yuntao Wu, Yongjun Jiang

**Affiliations:** ^1^NHC Key Laboratory of AIDS Immunology (China Medical University), National Clinical Research Center for Laboratory Medicine, The First Affiliated Hospital of China Medical University, Shenyang, China; ^2^Department of Clinical Laboratory, The Second Affiliated Hospital of Soochow University, Suzhou, China; ^3^National Center for Biodefense and Infectious Diseases, School of Systems Biology, George Mason University, Manassas, VA, United States

**Keywords:** CXCL10, resting memory CD4^+^ T cells, latent infection, HIV reservoir, cofilin

## Abstract

A major barrier to HIV eradication is the persistence of viral reservoirs. Resting CD4^+^ T cells are thought to be one of the major viral reservoirs, However, the underlying mechanism regulating HIV infection and the establishment of viral reservoir in T cells remain poorly understood. We have investigated the role of IP-10 in the establishment of HIV reservoirs in CD4^+^ T cells, and found that in HIV-infected individuals, plasma IP-10 was elevated, and positively correlated with HIV viral load and viral reservoir size. In addition, we found that binding of IP-10 to CXCR3 enhanced HIV latent infection of resting CD4^+^ T cells *in vitro.* Mechanistically, IP-10 stimulation promoted cofilin activity and actin dynamics, facilitating HIV entry and DNA integration. Moreover, treatment of resting CD4^+^ T cells with a LIM kinase inhibitor R10015 blocked cofilin phosphorylation and abrogated IP-10-mediated enhancement of HIV latent infection. These results suggest that IP-10 is a critical factor involved in HIV latent infection, and that therapeutic targeting of IP-10 may be a potential strategy for inhibiting HIV latent infection.

## Introduction

Persistence of viral reservoirs is the main barrier for curing human immunodeficiency virus (HIV) infection. Without persistent-suppression or elimination of viral reservoirs, HIV-infected individuals need lifelong antiretroviral therapy (ART) (1, 2). The “kick and kill” strategy may re-activate HIV from latent reservoirs, but may not be capable of effectively reducing viral reservoir size (3, 4). Elucidating the mechanisms by which HIV reservoirs are established are critical for the development of new therapeutic strategies for a cure or functional cure. Although the molecular mechanisms regulating the transcriptional reactivation of HIV latent reservoirs have been extensively explored, factors affecting HIV latent infection and the establishment of viral reservoirs in memory T cells remain poorly understood.

HIV reservoirs are established immediately during primary HIV infection, possibly as a result of direct infection of T cells that is facilitated by inflammatory chemokines released within the first 1‒2 weeks (5). Chemokines are small chemotactic cytokines that can induce chemotaxis of T cells; they are classified into 4 subfamilies (CXC, CC, CX3C, and XC) based on the number and spacing of conserved cysteine residues at the N terminus of the peptide chain (6, 7). In viral infection, levels of chemokines are elevated early, as a natural host antiviral response (8, 9). However, this early response is frequently exploited by viruses to facilitate infection and pathogenesis (10–12). Previous *in vitro* studies have shown that the CC family chemokines chemokine (C-C motif) ligand 19 (CCL19) and CCL21 promote HIV-1 provirus integration and latent HIV infection in resting memory CD4^+^ T cells, suggesting that they are directly involved in the establishment of viral reservoirs (13–16). Additionally, CCL2 is also found to stimulate HIV replication in T lymphocytes, macrophages, and peripheral blood mononuclear cells (17–19). However, our recent *in vivo* studies of HIV-infected patients have found that the aforementioned chemokines were not significantly upregulated following HIV infection, suggesting that these chemokines, although enhancing the establishment of viral latent reservoirs *in vitro*, may not be the major chemokines to promote the establishment of viral reservoirs *in vivo*. Other chemokines may also be involved, and could play important roles.

Interferon-γ‒inducible protein (IP)-10—also known as C-X-C motif chemokine ligand (CXCL)10—is mainly secreted by cluster of differentiation (CD)4^+^ T cells, CD8^+^ T cells, monocytes, and myeloid dendritic cells, and is elevated in multiple diseases (20, 21) as well as in HIV infection (22, 23). It has been reported that plasma IP-10 is associated with rapid disease progression in early HIV-1 infection (24). In addition, high-levels of IP-10 in plasma and small intestine are associated with more rapid HIV/SIV disease onset after infection (25). Moreover, IP-10 has also been found to inhibit T cell function in HIV-1-infected individuals on ART (26); during untreated, acute HIV infection, the virus replicative capacity positively correlated with IP-10 (27); the peak of plasma IP-10 level precedes that of HIV viremia (28), and is a strong predictor for peak viral load (29, 30). In addition, Cameron and co-authors have also showed that the presence of IP-10 led to high levels of viral integration and reverse transcription in resting CD4^+^ T cells (16). Nevertheless, although a stimulatory effect of IP-10 on HIV infection is certain, the direct role of IP-10 on viral early infection steps such as viral entry and integration as well as the establishment of viral reservoir was not completely clear and needs to be further elucidated.

HIV latent infection is thought to be established in 2 ways. Firstly, HIV can infect activated CD4^+^ T cells, and a majority of these infected cells can be killed by the virus or eliminated by antiviral immunity; surviving cells undergo effector-to-memory transition to a resting state and persist over the long term (31, 32). Secondly, HIV can also directly infect resting memory CD4^+^ T cells, resulting in latent infection (33, 34). Latent provirus does not produce viral proteins, minimizing virus-induced cytopathic effects and evading immune clearance. Consistent with the model of direct infection of blood resting T cells, it has been shown that HIV glycoprotein (gp)120 binds to the C-X-C chemokine receptor (CXCR)4 can activate Gαi-dependent signaling, which promotes cofilin activation and actin dynamics to overcome the barrier of actin cytoskeleton in resting CD4^+^ T cells (10). In addition, Cameron et al. have also demonstrated that the establishment of HIV latent infection in CD4^+^ T cells was promoted by CCL19/21-mediated increases of actin dynamics *via* the cofilin pathway (16).

The cortical actin underneath the plasma membrane provides the structural support of cells, and is the driving force for T cell motility (35, 36). During inflammation and infection, chemokines are released to reorganize the filamentous actin (F-actin) cytoskeleton for cell migration along the chemokine concentration gradient (37). The actin-depolymerizing proteins, cofilin, plays a major role in modulating the actin dynamics (38, 39).

Given that cofilin-induced actin dynamics plays an important role in chemokine-mediated enhancement of HIV latent infection of T cells, we hypothesized that IP-10-mediated enhancement of HIV infection may involve the cofilin pathway. In this article, we tested our hypothesis by investigating the relationship between the plasma IP-10 level, viral load, and intracellular HIV DNA. We also studied effects of IP-10 on HIV infection of resting CD4^+^ T cells, and IP-10-initiated actin dynamics and cofilin signaling. Our results demonstrate that IP-10 is a key factor involved in the establishment of HIV latency in resting CD4^+^ T cells.

## Methods and Details

### Participants and Sample Collection

HIV^+^ participants were recruited from the men who have sex with men cohort of Key Laboratory of AIDS Immunology of the National Health and Family Planning Commission at The First Affiliated Hospital of China Medical University (CMU). HIV negative controls (NCs) were recruited from the voluntary HIV counseling and testing center of CMU and had no apparent active infections including hepatitis B or C infection. All participants provided written, informed consent at the time of blood sample collection. The study was approved by The Research and Ethics Committee of CMU and conducted according to the principles outlined in the Declaration of Helsinki. A total of 28 HIV^+^ ART-naïve participants and 24 age- and sex-matched NCs were enrolled. The characteristics of the study population are summarized in **Table 1**. Samples from NCs were used in viral infection experiments. Quantity of HIV intracellular DNA were evaluated in 29 ART-treated HIV^+^ participants who were classified into 2 groups according to plasma IP-10 levels: high IP-10 (>1000 pg/ml) after receiving ART for over 20 months (median, 30 months); and low IP-10 (≤1000 pg/ml) after receiving ART for at least 24 months (median, 28.5 months). All participants had undetectable viral load at the time of enrollment (detection limit for viral load: 20 copies/ml). CD4^+^ T cell count and viral load were measured every 3 months, and none of the HIV^+^ participants had apparent active opportunistic infections at the time of the study. Clinical parameters of the participants are listed in **Table S3**.

**Table 1:** Characteristics of study participants.

	HIV^+^ (n=28)	HC (n=24)	*P* value*
*Median age (range), years	28 (19–54)	31 (21-50)	>0.05
Sex, no. (%)	Male: 28 (100)	Male: 20 (83)	>0.05
		Female: 4 (17)	
HIV subtype (n)			
CRF01-AE	16		
CRF07-BC	4		
URF01-B	1	NA	NA
URF01-C	1		
B	2		
ND	4		
Median CD4^+^ T cell count (range), cells/mm³	286 (38–913)	676 (396–1342)	<0.05
Median viral load (range), copies/ml	52,700 (331–1,210,000)	NA	NA

All HIV subjects were antiretroviral therapy-naïve.

*HIV^+^ vs HC.

CD4, cluster of differentiation 4; HC, health control; HIV^+^, human immunodeficiency virus-positive; NA, not applicable; ND, not determined.

### Plasma Chemokine/Cytokine Detection

Plasma chemokine/cytokine levels were analyzed in 52 plasma samples from HIV^+^ ART-naïve participants (n=28) and NCs (n=24) using the Bio-Plex Pro Human Chemokine Panel 40-Plex (Bio-Rad, Hercules, CA, USA), which detected 6Ckine/CCL21, B cell-attracting chemokine (BCA)-1/CXCL13, cutaneous T-cell-attracting chemokine/CCL27, epithelial-derived neutrophil-activating peptide 78/CXCL5, Eotaxin/CCL11, Eotaxin-2/CCL24, Eotaxin-3/CCL26, Fractalkine/CX3CL1, granulocyte chemotactic protein (GCP)-2/CXCL6, granulocyte-macrophage colony-stimulating factor, Gro-a/CXCL1, Gro-b/CXCL2, I-309/CCL1, interferon-γ, interleukin (IL)-1b, IL-2, IL-4, IL-6, IL-8/CXCL8, IL-10, IL-16, IP-10/CXCL10, interferon-inducible T-cell alpha chemoattractant (I-TAC)/CXCL11, monocyte chemoattractant protein (MCP)-1/CCL2, MCP-2/CCL8, MCP-3/CCL7, MCP-4/CCL13, macrophage-derived chemokine (MDC)/CCL22, macrophage migration inhibitory factor, monokine induced by gamma interferon (MIG)/CXCL9, macrophage inflammatory protein (MIP)-1a/CCL3, MIP-1s/CCL15, MIP-3a/CCL20, MIP-3b/CCL19, myeloid progenitor inhibitory factor 1/CCL23, small inducible cytokine (SCY)B16/CXCL16, stromal cell-derived factor 1a+b/CXCL12, thymus and activation regulated chemokine (TARC)/CCL17, thymus-expressed chemokine/CCL25, and tumor necrosis factor (TNF)-α. Chemokine/cytokine standards supplied by the manufacturer were detected in duplicate on each array. Data were acquired using a Bio-Plex 200 System (Bio-Rad). Chemokine/cytokine levels below/above the minimum/maximum detection thresholds are reported as minimum/maximum threshold values.

### Isolation of Resting Memory CD4^+^ T Cells From Peripheral Blood

Peripheral blood mononuclear cells (PBMCs) freshly obtained from subjects were purified by Ficoll-Hypaque density gradient centrifugation, and resting memory CD4^+^ T cells were purified through 2 rounds of negative selection as previously described (40, 41). Briefly, monoclonal antibodies against human CD14, CD56, human leukocyte antigen (HLA)-DR/DP/DQ, CD8, CD11b, CD19, CD45RA, CD69, and CD25 were used. Antibody-bound cells were depleted using Dynabeads Pan Mouse IgG (Thermo Fisher Scientific, Waltham, MA, USA). Purified cells were cultured in Roswell Park Memorial Institute (RPMI)1640 medium supplemented with 10% heat-inactivated fetal bovine serum (FBS), penicillin (50 U/ml), and streptomycin (50 g/ml) (all from Invitrogen, Carlsbad, CA, USA). The cells were cultured overnight before infection or treatment.

### Virus Preparation and Infection of Resting Memory CD4^+^ T Cells

Virus stocks of HIV-1 clones NL4-3 and AD8 were prepared by transfection of human embryonic kidney 293T cells with cloned proviral DNA as previously described (10, 42). The supernatant was harvested 48 h later and p24 level in the viral supernatant was measured in triplicate by enzyme-linked immunosorbent assay (ELISA) using the P24 antigen ELISA kit (QuantoBio, Beijing, China). Before infection, resting memory CD4^+^ T cells were treated with recombinant human IP-10 (5 ng/ml), human anti-IP-10 monoclonal antibody (mAb) (200 ng/ml), or the CXCR3 antagonist (±)-NBI 74330 (500 pg/ml) (all from R&D Systems, Minneapolis, MN, USA); jasplakinolide (Jas) (500 nM; CalBiochem, San Diego, CA, USA); or the LIM domain kinase (LIMK) inhibitor R10015 (100 μM; Virongy, Manassas, VA, USA) for 1 h. For NL4-3 infection, the treated resting memory CD4^+^ T cells were incubated with NL4-3/AD8 for 2 h and then washed twice with medium to remove free virus. The reagents (IP-10, IP-10 mAb, (±)-NBI 74330, Jas, and R10015) were added back to the infected cells, which were resuspended in fresh RPMI-1640 medium supplemented with 10% FBS, penicillin (50 U/ml), and streptomycin (50 g/ml) at a density of 10^6^/ml, followed by incubation for 5 days without stimulation. At the 5th day, cells were activated for 9 days with anti-CD3/CD28 magnetic beads (Invitrogen) at the concentration recommended by the manufacturer (for a total culture time of 14 days) (**Figure 2B**). In additional experiment, IP-10 was washed out at the 5th day, and then cells were activated for 9 days with anti-CD3/CD28 magnetic beads (Invitrogen) at the concentration recommended by the manufacturer (for a total culture time of 14 days) (**Figure 3A**).

For the viral replication assay, 200 μl of culture supernatant was collected on days 1, 3, 5, 7, 9, 12, and 14 after infection and stored at −135°C for p24 ELISA. The level of p24 in the supernatant was measured using a P24 antigen ELISA kit (QuantoBio). The absorbance at 630 nm was measured using an ELx808 automatic microplate reader (Bio-Tek, Winooski, VT, USA).

### Evaluation of Activation Markers on Resting Memory CD4^+^ T Cells by Flow Cytometry

The activation markers expression on resting memory CD4^+^ T cells were detected after IP-10 or CD3/CD28 stimulated and measured using the following fluorophore-conjugated antibodies: phycoerythrin (PE)-Cy7 anti-human CD3, allophycocyanin (APC)-Cy7 anti-human CD4, peridinin-chlorophyll-protein anti-human CD197/CCR7, fluorescein isothiocyanate (FITC) anti-human CD45RA, APC anti-human CD69, APC anti-human CD25, APC anti-human HLA-DR, APC mouse IgG1 κ isotype control, and PE mouse IgG1 κ isotype control (all from Biolegend). Samples were analyzed using an LSR II flow cytometer (BD Biosciences), which was adjusted with 10-peak color rainbow beads (Spherotech, Lake Forest, IL, USA). Gates were defined using appropriate isotype controls. Expression levels in each sample were analyzed with FlowJo v10 software (Ashland, OR, USA).

### Measurement of F-Actin by Flow Cytometry

Resting memory CD4^+^ T cells were cultured overnight in medium before treatment with IP-10 at 37°C for up to 60 min. Actin dynamics were evaluated at 1, 5, 10, 15, 30, 45, and 60 min, with the measurement at 0 min serving as the reference value. Data are expressed as the percentage of F-actin. After treatment of resting memory CD4^+^ T cells with IP-10 (5 ng/ml), cells were washed with cold phosphate-buffered saline (PBS) with 0.1% bovine serum albumin and resuspended in BD Cytofix/Cytoperm Solution (BD Biosciences) for 30 min at 4°C, then washed twice with BD Perm/Wash Buffer (BD Biosciences). F-actin was labeled by treatment with FITC-labeled phalloidin (Sigma-Aldrich, St. Louis, MO, USA) for 30 min and detected by flow cytometry; untreated cells served as the background control. The cells were washed twice with BD Perm/Wash Buffer and fixed with 1% paraformaldehyde, then analyzed on an LSR-II flow cytometer (BD Biosciences).

### Measurement of Phosphorylated Cofilin by Flow Cytometry

Cofilin dynamics were measured as the percentage of phospho- (p-)cofilin. Resting memory CD4^+^ T cells were treated with IP-10 at 1, 5, 10, 15, 30, 45, and 60 min, then fixed with 1.5% formaldehyde for 10 min, permeabilized with cold (−20°C) MeOH, and stained with a rabbit anti‒p-cofilin (Ser3) antibody (Cell Signaling Technology, Danvers, MA, USA) for 1 h at room temperature. The cells were washed and labeled with Alexa Fluor 488-conjugated chicken anti-rabbit antibody (Invitrogen) for 30 min at room temperature, then analyzed on an LSR-II flow cytometer (BD Biosciences).

### Measurement of HIV Entry Into Resting Memory CD4^+^ T Cells

A β-lactamase (BlaM) substrate CCF2-based system was used to evaluate the role of IP-10 in HIV entry. CCF2 dye is a fluorescent substrate of BlaM that can be loaded in multiple cells; the fluorescence emission spectrum of CCF2 changes from green (520 nm) to blue (447 nm) when the β-lactam ring is cleaved by BlaM. The change in emission wavelength allows estimation of the percentage of HIV-infected cells by flow cytometry (43).

For the production of BlaM-Vpr‒containing NL4-3 virions, pNL4-3 proviral DNA (60 μg) and pCMV-Blam-Vpr (20 μg) and pAdVAntage vectors (10 μg) were added to 293T cells grown in a 100-mm culture dish and cultured for 48 h at 37°C; the culture supernatant was collected and centrifuged at low speed at 4°C. The virion-enriched supernatant was stored as aliquots at −80°C until use.

To evaluate the effect of IP-10 on HIV entry, IP-10 (5 ng/ml) was added to the culture medium of resting memory CD4^+^ T cells for 1 h at 37°C; the cells were then incubated with BlaM-Vpr-containing NL4-3 virion for 2 h at 37°C, washed in CO_2_-independent medium (Gibco, Grand Island, NY, USA), and loaded with 2 μl of 1 mM CCF2/AM dye (Invitrogen) in loading buffer (8 μl of 0.1% acetic acid containing 100 mg/ml Plironic-F127R, and 1 ml of CO_2_-independent medium) for 1 h at room temperature according to the manufacturer’s instructions. After 2 washes with CO_2_-independent medium, the BlaM reaction was allowed to proceed for 7 h at room temperature in 200 μl CO_2_-independent medium containing 10% FBS, 50 U/ml penicillin, and 50 g/ml streptomycin. The cells were washed once in PBS and fixed in PBS with 1.2% paraformaldehyde, then analyzed by flow cytometry.

### Analysis of HIV DNA Integration

The effect of IP-10 on the integration of HIV DNA was measured *in vitro* by Alu-PCR and *in vivo* by digital droplet (dd)PCR. Resting memory CD4^+^ T cells were evenly distributed into the following 5 groups: negative control (untreated), NL4-3 (treated with NL4-3), NL4-3+IP-10 (treated with NL4-3 and 5 ng/ml IP-10), NL4-3+raltegravir (treated with NL4-3 and 50 μM raltegravir), and NL4-3+IP-10+raltegravir (treated with 5 ng/ml IP-10, NL4-3, and 50 μM raltegravir). IP-10 and raltegravir were added for 1 h before NL4-3 infection. The cells were cultured for 4 days with the reagents, which were replenished every 2 days, then collected on day 4 for DNA extraction using the QIAamp DNA Blood Mini Kit (Qiagen, Valencia, CA, USA). Quantitative PCR was performed on the LightCycler 480 (Roche, Basel, Switzerland) to detect integrated HIV DNA. The primers and probes used for the 2 rounds of amplification are shown in **Table S1**. Ribonuclease P/MRP Subunit (RP)P30 was used as the reference gene. The reaction conditions for round 1 were as follows: initial denaturation at 94°C for 2 min and 68°C for 10 min, followed by 12 amplification cycles (94°C for 15 s, 60°C for 30 s, and 68°C for 4 min) and holding at 4°C in the final step. The reaction conditions for round 2 were as follows: 50°C for 2 min and 95°C for 10 min, followed by 40 amplification cycles (95°C for 15 s and 60°C for 1 min).

To analyze the relationship between early IP-10 level and HIV intracellular DNA, 29 ART-treated participants were divided into 2 groups according to IP-10 level at the primary infection stage (**Supplementary Table S3**). As viral load decreases rapidly after ART and is barely detectable after 6 months, HIV DNA level in PBMCs is used as a measure of HIV reservoir size. DdPCR is sensitive enough to detect small quantities of HIV DNA in cells. PBMCs obtained from participants after ART and serum samples from ART-naïve participants were stored in liquid nitrogen. IP-10 level was measured using the Bio-Plex Pro Human Cytokine 27-plex (Bio-Rad). DNA was extracted from PBMCs using the QIAamp DNA Blood Mini Kit (QIAGEN). HIV reservoir size was measured with the QX200 Droplet Digital PCR System (Bio-Rad), with RPP30 serving as the reference gene. The primers and probes that were used are shown in **Supplementary Table S2**. The reaction conditions were as follows: initial denaturation at 95°C for 10 min and then at 98°C for 10 min, followed by 50 amplification cycles (94°C for 30 s and 62°C for 1 min) and holding at 16°C in the final step.

### Statistical Analysis

Statistical analysis was performed using Prism v6.0 (GraphPad, La Jolla, CA, USA) and SPSS Statistics v20.0 (SPSS Inc, Chicago, IL, USA) software programs. Normality tests were performed before analyzing the data. The nonparametric Mann–Whitney U test was used to evaluate differences between 2 groups. The Spearman rank correlation test was used to estimate the correlation between chemokine level and viral load. One-way analysis of variance was used to compare P24 levels between 4 groups in the signaling pathway experiment. All tests were 2-tailed and *P* values <0.05 were considered significant.

## Results

### Elevated IP-10 Level During HIV Infection Is Positively Correlated With HIV Viral Load and HIV Intracellular DNA After ART

**Figure 1:**
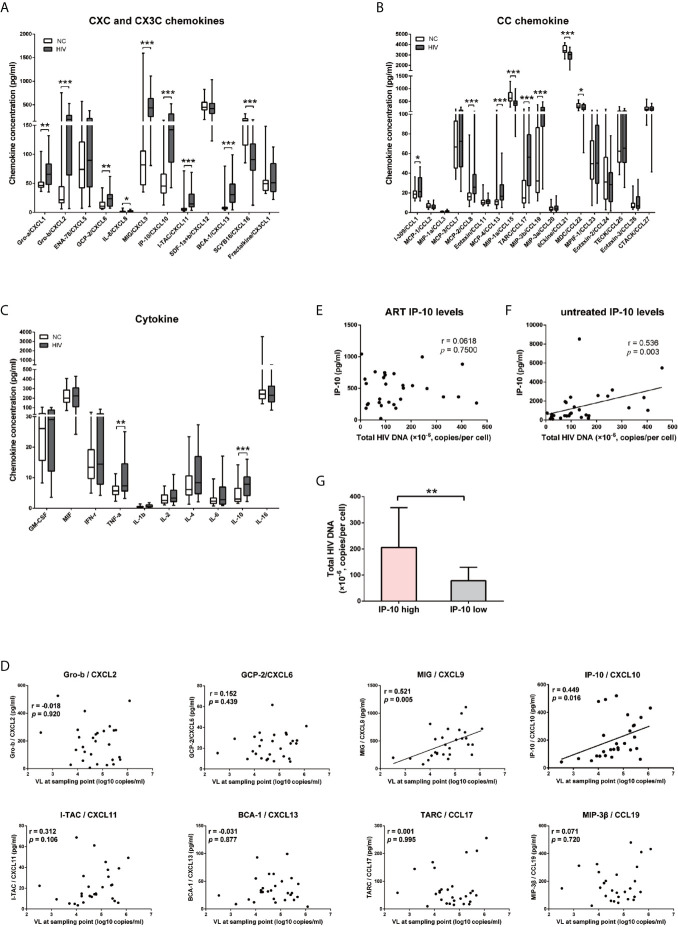
IP-10/CXCL10 level is elevated in the chemokine profile of HIV^+^ participants, and positively correlated with viral load and HIV reservoir size. **(A–C)** Comparison of 11 CXC chemokines and 1 CX3C chemokine **(A)**; 8 CC chemokines **(B)**; and 10 cytokines **(C)** between HIV+ participants (gray bars, n=28) and NCs (white bars, n=24). Graphs show data as median ± interquartile range. The nonparametric Wilcoxon matched-pairs test was used for intergroup comparisons. **(D–F)** Spearman correlation analyses of chemokine (Gro-b/CXCL2, GCP-2/CXCL6, MIG/CXCL9, IP-10/CXCL10, I-TAC/CXCL11, BCA-1/CXCL13, TARC/CCL17, and MIP-3β/CCL19) levels and viral load at indicated time points **(D)**; and between total HIV DNA of PBMCs and IP-10 level after ART **(E)** or without treatment **(F)**. **(G)** Comparison the total HIV DNA of PBMCs in IP-10 high vs IP-10 low groups (separated according to median IP-10 level in 29 ART-treated HIV^+^ participants). The nonparametric Mann–Whitney U test was used for intergroup comparisons (IP-10 high: N=13; IP-10 low: N=16, *P*=0.001). *P < 0.05, **P < 0.01, ***P < 0.001.

Previous studies have reported that the releases of cytokine and chemokine before the peak of viremia (5, 44) and the establishment of viral reservoirs are early events in HIV infection (45, 46). We speculated that early cytokine/chemokine induction may impact HIV replication and the establishment of viral reservoirs. To test this hypothesis, we compared the levels of 40 cytokines and chemokines in participants with primary HIV infection (HIV^+^, n=28) and NCs (n=24) (**Table 1**), and found that 19 were significantly altered (15 upregulated and 4 downregulated in HIV^+^ vs NCs); of these, 9 were CXC chemokines (**Figure 1A**), 8 were CC chemokines (**Figure 1B**), and 2 were cytokines (**Figure 1C**). Only IP-10/CXCL10 (*P*=0.005) and MIG/CXCL9 (*P*=0.016) levels were positively correlated with early viral load (**Figure 1D**), with no significant correlation observed for the other 15 chemokines and 2 cytokines (**Figure 1D** and **Supplementary Figure S1**). In a comparison of median chemokine levels between HIV^+^ and NCs, IP-10 showed large difference (213.81%) (data not shown), consistent with our previous studies (47).

Given the significantly elevated level of IP-10 in HIV infection, we examined the correlation between plasma level of IP-10 and HIV DNA level in PBMCs of 29 HIV-1–infected participants who received ART for 30 months (**Table S3**). There was no correlation between IP-10 level after ART and HIV DNA copy number in PBMCs (**Figure 1E**); however, the pre-ART IP-10 level was correlated with post-ART HIV DNA copy number (**Figure 1F**). We divided the 29 ART-treated participants into 2 groups according to plasma IP-10 level before ART—i.e., IP-10 high (IP-10 >1000 pg/ml) and IP-10 low (IP-10 <1000 pg/ml). We then measured HIV genomic DNA level in PBMCs of these participants after ART, and found that the level was higher in the IP-10 high group than in the IP-10 low group (**Figure 1G**). These results suggest that IP-10 might be a key factor promoting HIV infection in peripheral blood.

### IP-10 Binds to CXCR3 to Promote HIV Latency Through Direct Infection but Not Activation of Resting Memory CD4^+^ T Cells

**Figure 2:**
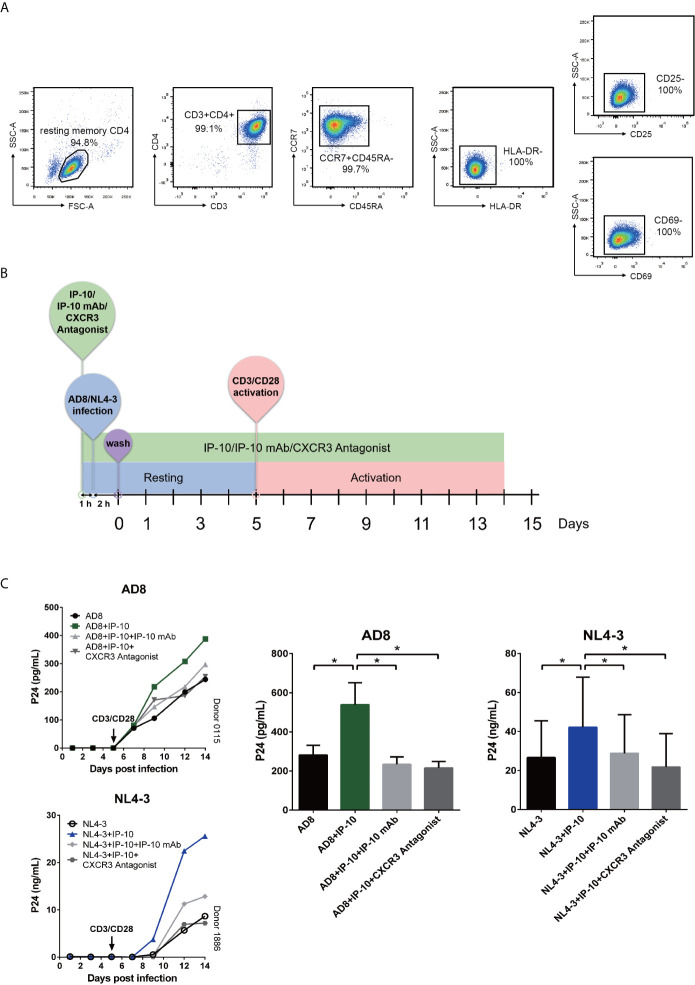
IP-10 promotes HIV AD8 and NL4-3 infection of resting memory CD4^+^ T cells. **(A)** Gating strategy for isolating a pure population of resting memory CD4^+^ T cells. **(B)** Schematic illustration of the procedure for evaluating the role of IP-10 in HIV infection of resting memory CD4^+^ T cells. **(C)** Kinetics of the effect of AD8 (n = 6)/NL4-3(n = 11), AD8/NL4-3+IP-10, AD8/NL4-3+IP-10+mAb, and AD8/NL4-3+IP-10+CXCR3 on resting memory CD4^+^ T cell from 2 representative NCs. P24 levels were compared between groups on the 14^th^ day post infection by one-way analysis of variance. *P < 0.05.

We next examined whether IP-10 plays a role in HIV infection of resting memory CD4^+^ T cells. Cells were isolated using magnetic beads at a purity of >94% (**Figure 2A**). Resting memory CD4^+^ T cells were latently infected with the R5-tropic AD8 or X4-tropic NL4-3 HIV strain for 5 days in the presence or absence of IP-10, then activated on day 5 with CD3/CD28 beads to stimulate productive viral replication (**Figure 2B**). Latent viral infection was enhanced by IP-10 in cells from multiple donors infected with either AD8 (*P*=0.001) or NL4-3 (*P*=0.031) (**Figure 2C**).

**Figure 3:**
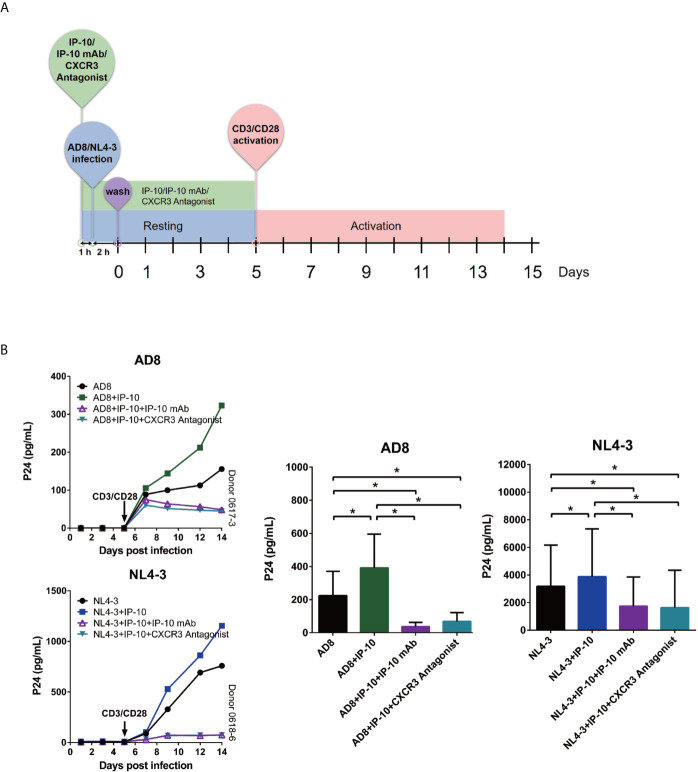
IP-10 promotes HIV AD8 and NL4-3 infection of resting memory CD4^+^ T cells at the resting state. **(A)** Schematic illustration of the procedure for evaluating the role of IP-10 in HIV infection of resting memory CD4^+^ T cells at the resting state. **(B)** Kinetics of the effect of AD8(n = 7)/NL4-3(n = 8), AD8/NL4-3+IP-10, AD8/NL4-3+IP-10+mAb, and AD8/NL4-3+IP-10+CXCR3 on resting memory CD4^+^ T cell at resting state from 2 representative NCs. P24 levels were compared between groups on the 14^th^ day post infection by one-way analysis of variance. *P < 0.05.

**Figure 4:**
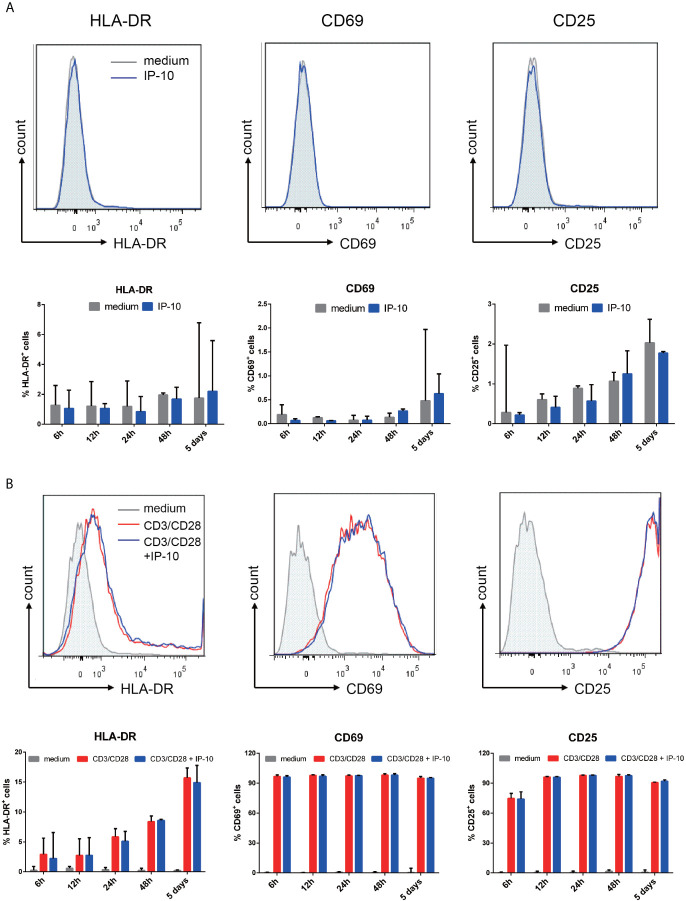
IP-10 does not promote resting memory CD4^+^ T cell activation. **(A, B)** Effect of IP-10 on the expression of the resting memory CD4^+^ T cell activation markers HLA-DR, CD69, and CD25 **(A)** and on the activation of resting memory CD4^+^ T cells by CD3/CD28 **(B)** at 6, 12, 24, and 48 h and 5 days post-treatment; representative flow cytometry plots from 3 participants and quantitative analyses are shown. The time point for HLA-DR, CD69, and CD25 of the representative flow cytometry plots are 5 days, 6 h and 24 h, respectively. The nonparametric Wilcoxon matched-pairs test was used for intergroup comparisons.

We examined whether the enhancement of latent HIV infection by IP-10 can be blocked by anti-IP-10 mAb or the CXCR3 antagonist (±)-NBI 74330. Resting CD4^+^ T cells were treated with these reagents along with IP-10 for 1 h before infection and activation (**Figure 2B**). Both anti-IP-10 mAb (AD8: *P*=0.016; NL4-3: *P*=0.031) and CXCR3 antagonist (AD8: *P*=0.031; NL4-3: *P*=0.031) abrogated the IP-10-mediated enhancement of HIV latent infection (**Figure 2C**). Thus, IP-10 promotes HIV latent infection of resting memory CD4^+^ T cells through binding with CXCR3. To exclude the possibility that IP-10 present in the culture medium may enhance HIV infection through promoting T cell activation, we conducted additional experiments by removing IP-10 at 5 days post infection prior to T cell activation (**Figure 3A**). We observed similar enhancement of HIV infection by IP-10 (**Figure 3B**), excluding the possibility that IP-10 enhances HIV infection indirectly by enhancing T cell activation. In addition, we also examined whether IP-10 can directly activating resting memory CD4^+^ T cells and found that multiple T-cell activation markers including CD69, CD25, and HLA-DR were not upregulated in resting memory CD4^+^ T cells after IP-10 treatment (**Figure 4A**). As CD3/CD28 was used to activate resting memory CD4^+^ T cells in the experiment, we tested whether IP-10 would potentiate the effect of CD3/CD28 to promote HIV infection and determined that it can’t enhance the activation of resting memory CD4^+^ T cells induced by CD3/CD28 (**Figure 4B**). We also examined the effect of IP-10 on T cell proliferation and apoptosis and found that IP-10 had no detectable effect on violet distribution and the proportion of Annexin V and 7-amino-actinomycin D–positive cells (**Figure S2**). These results suggest that IP-10 promotes HIV latency likely by directly acting on HIV infection steps and not by activating resting memory CD4^+^ T cells.

### IP-10 Promotes Actin Dynamics and HIV Entry Into Resting Memory CD4^+^ T Cells and Viral Integration

**Figure 5:**
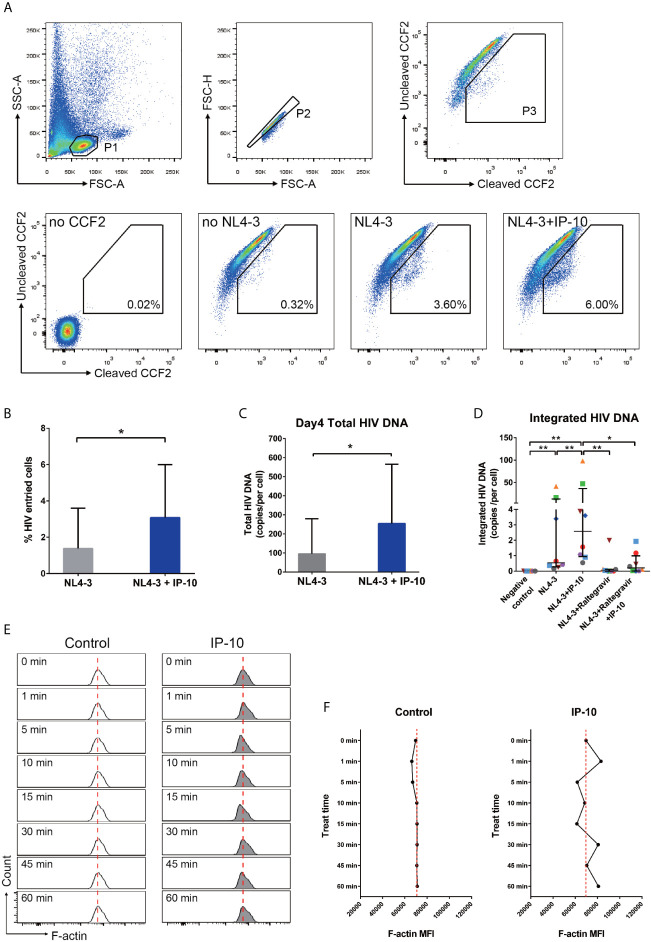
IP-10 promotes HIV NL4-3 entry into resting memory CD4^+^ T cells, viral DNA synthesis and integration, and actin activation. **(A)** Gating strategy and representative flow cytometry plots from the experiment evaluating the role of IP-10 in the infection of resting memory CD4+ T cells by HIV NL4-3. Viral entry into resting memory CD4+ T cells was evaluated with the BlaM-Vpr entry assay. For *no CCF2*, cells were pretreated for 1 h with IP-10 (5 ng/ml) and then infected with NL4-3. For *no NL4-3*, cells were treated with CCF2 without NL4-3 infection. For *NL4-3*, cells were treated with CCF2 before infection with NL4-3. For *NL4-3+IP-10*, cells were treated with CCF2 before pretreatment for 1 h with IP-10 (5 ng/ml), and then infected with NL4-3. The Wilcoxon matched-pairs test was used to compare median values between two groups (N=7, *P*=0.016). **(B)** Quantification of HIV+ cells in NL4-3 and NL4-3+IP-10 groups. The Wilcoxon matched-pairs test was used to compare median values between groups. **(C)** For *NL4-3*, cells were infected with NL4-3; *for NL4-3+IP-10*, cells were pretreated for 1 h with IP-10 (5 ng/ml) and then infected with NL4-3. Cells were infected for 2 h, washed, and then collected at different time points for ddPCR quantification of total viral DNA at 4 days. Results are expressed as copies/cell. The Wilcoxon matched-pairs test was used to compare median values between groups (N=6, *P*=0.031). **(D)** Quantitative (Alu) PCR analysis of HIV DNA integration in negative control, NL4-3, NL4-3+IP-10, NL4-3+raltegravir, and NL4-3+raltegravir+IP-10 groups. As the variances are different, the Wilcoxon matched-pairs test was used to compare median values between groups (N=8, NL4-3 vs. NC: *P*=0.008; NL4-3+IP-10 *vs*. NC: *P*=0.008; NL4-3+IP-10 *vs*. NL4-3: *P*=0.008; NL4-3+Raltegravir *vs*. NL4-3+IP-10: *P*=0.008; NL4-3+Raltegravir +IP-10 *vs.* NL4-3+IP-10: *P*=0.039; NL4-3+Raltegravir *vs.* NC: *P*=0.062; NL4-3+Raltegravir +IP-10 *vs*. NC: *P*=0.016; NL4-3+Raltegravir vs. NL4-3: *P*=0.078; NL4-3+Raltegravir+IP-10 *vs.* NL4-3+Raltegravir: *P*=0.469). **(E)** Representative flow cytometry plots from the experiment evaluating the effect of IP-10 on actin polymerization and depolymerization over time. The red dashed line represents localized baseline unstimulated F-actin level. **(F)** Temporal profile of MFI in control and IP-10–treated resting memory CD4^+^ T cells (The result for one of the three separated experiments is showed in the figure). *P < 0.05, **P < 0.01.

To investigate the mechanism underlying IP-10-mediated enhancement of latent HIV infection of resting memory CD4^+^ T cells, we performed stepwise mapping of the HIV infection time course in the presence of IP-10. We first quantified viral entry using the BlaM-Vpr entry assay (43), and found that IP-10 pretreatment of resting memory CD4^+^ T cells increased viral entry (NL4-3+IP-10 vs NL4-3; *P*=0.016) (**Figures 5A, B**). We then quantified post-entry viral DNA synthesis and integration and observed increases in viral DNA synthesis (*P*=0.031) (**Figure 5C**) and integration (*P*=0.008) (**Figure 5D**) in IP-10 treated cells. Blocking HIV integration using the HIV integrase inhibitor raltegravir abolished the increase in viral integration induced by IP-10 (*P*=0.039) (**Figure 5D**). These results demonstrate that IP-10 promotes HIV entry, integration, and DNA synthesis in resting memory CD4^+^ T cells.

Static cortical actin in resting CD4^+^ T cells is a major barrier for latent HIV infection of resting CD4^+^ T cells (10, 48–53). HIV uses the envelope protein gp120 to initiate chemotactic signaling from chemokine coreceptor to increase actin dynamic, which is necessary for viral entry and nuclear migration. Given that IP-10 binding to CXCR3 also activates chemotactic signaling (54, 55), we speculated that IP-10 enhances viral infection through similar signaling with HIV gp120. To test this hypothesis, we analyzed the time course of actin polymerization in resting memory CD4^+^ T cells following IP-10 treatment and found that IP-10 triggered a transient increase in actin polymerization and depolymerization (**Figure 5E**), which was confirmed by a higher mean fluorescence intensity (MFI) of F-actin in IP-10–treated cells relative to control cells (**Figure 5F**).

### IP-10 Activates LIMK–Cofilin Signaling in Resting Memory CD4^+^ T Cells

**Figure 6:**
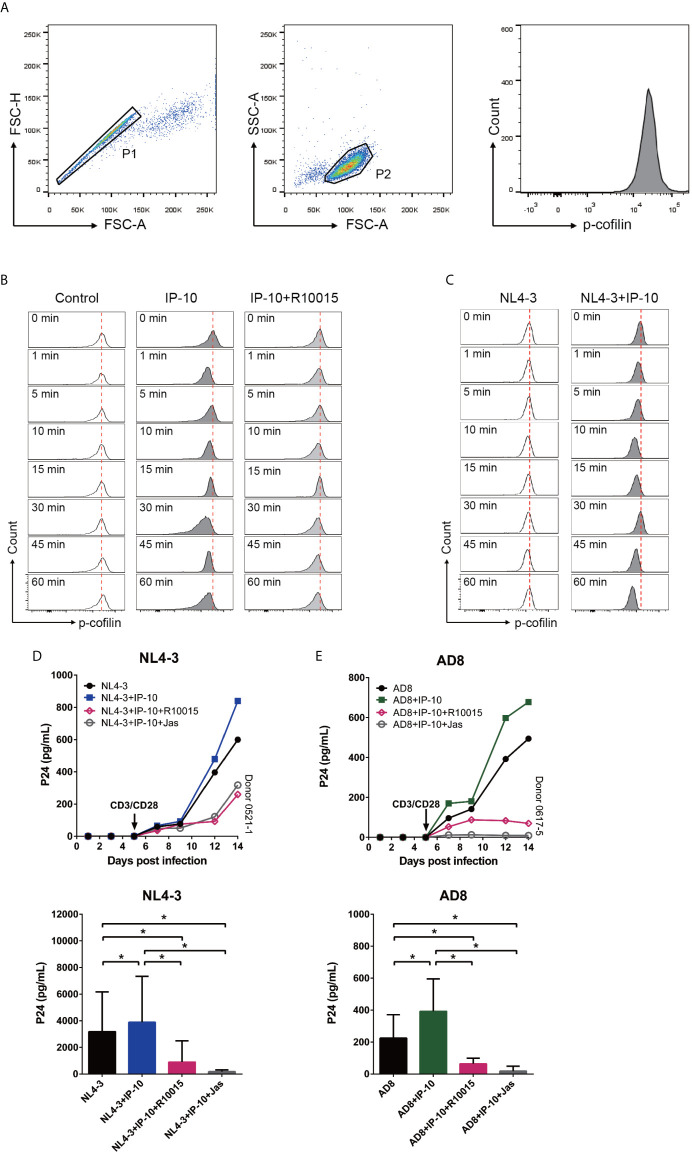
IP-10 activates LIMK–cofilin signaling in resting memory CD4^+^ T cells. **(A)** Gating strategy for the experiment evaluating the effect of IP-10 on cofilin activation. **(B, C)** Representative flow cytometry plots from the experiment evaluating the effect of IP-10 on cofilin phosphorylation over time. Resting memory CD4^+^ T cells were treated with IP-10 (5 ng/ml) and IP-10 (5 ng/ml)+R10015 (100 μM) **(B)** or NL4-3 and NL4-3+IP-10 (5 ng/ml) **(C)** for the indicated times, and p-cofilin was marked with Ser3, followed by flow cytometry analysis. The red dashed lines represent localized baselines for cofilin phosphorylation. Cofilin dynamics were evaluated at 1, 5, 10, 15, 30, 45, and 60 min, with the measurement at 0 min serving as the reference value. **(D, E)** LIMK inhibition abrogates the enhancement of HIV AD8/NL4-3 infection by IP-10. Representative plots of 2 participants are shown. P24 levels of day 14 were compared between groups by one-way analysis of variance (NL4-3: n = 7, AD8: n = 8). *P < 0.05.

The rearrangement of cortical actin, including its assembly and disassembly, is primarily regulated by the actin-depolymerizing factor cofilin (56). To clarify the involvement of cofilin in IP-10–mediated actin dynamics, we treated resting memory CD4^+^ T cells with IP-10 and analyzed the time course of cofilin phosphorylation (**Figure 6A**). Similar to its effect on actin, IP-10 caused rapid and cycles of cofilin phosphorylation/dephosphorylation after IP-10 treatment (**Figure 6B**). To determine whether the increase in cofilin phosphorylation induced by IP-10 was mediated through the cofilin kinase LIM domain kinase (LIMK), cells were treated with the LIMK inhibitor R10015 (40), which reduced p-cofilin level (**Figure 6B**). Thus, IP-10 facilitates latent HIV infection of resting memory CD4^+^ T cells by inducing cofilin activation and stimulating actin dynamics. Given that HIV-1 gp120 is known to trigger cofilin activation (57), we investigated whether IP-10 could promote HIV-mediated cofilin activity. Compared to IP-10-untreated, HIV (NL4-3)-infected resting memory CD4^+^ T cells, those that were both infected and treated with IP-10 showed increased cofilin phosphorylation/dephosphorylation (**Figure 6C**), indicating that IP-10 increases cofilin activity and actin dynamics through activation of the LIMK–cofilin pathway in synergy with HIV gp120-mediated cofilin activation.

To confirm that IP-10-mediated activation of cofilin and actin dynamics are related to its enhancement of latent HIV infection of resting memory CD4*^+^* T cells, we blocked IP-10-mediated enhancement of HIV infection using R10015 and the actin inhibitor Jas, respectively (58). Both R10015 and Jas significantly reduced the enhancement of latent HIV infection of resting memory CD4^+^ T cells by IP-10 (**Figures 6D, E**). These results provide evidence that IP-10–mediated cofilin activation promotes latent HIV infection of memory CD4^+^ T cells.

## Discussion

**Figure 7:**
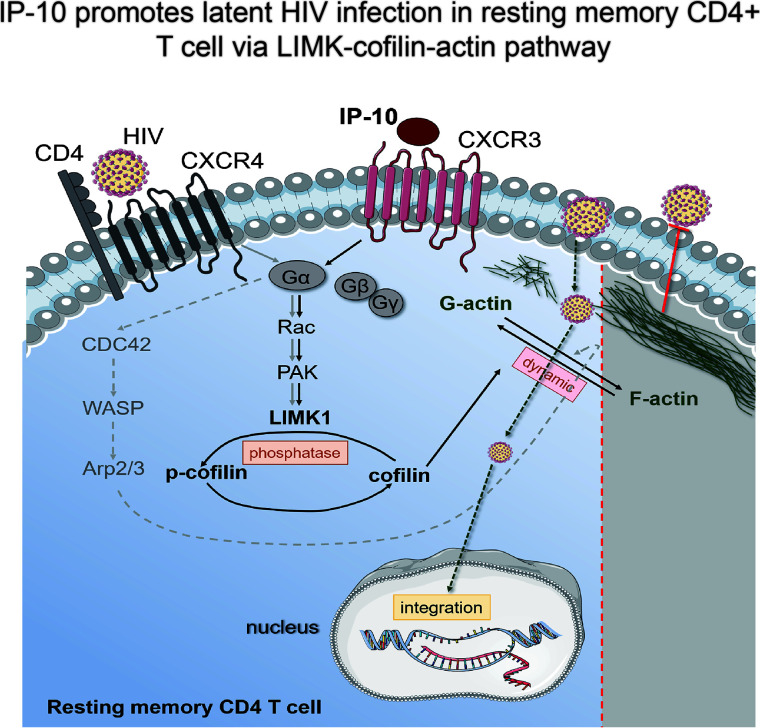
Model of IP-10-induced enhancement of latent HIV infection and reservoir establishment in resting memory CD4^+^ T cells. A signaling pathway common to HIV (gray solid line) and IP-10 (black solid line) induces actin activation. IP-10 binds to CXCR3 expressed on the surface of resting memory CD4^+^ T cells; Rac stimulates p21-activated kinase (PAK), which activates LIMK, leading to cofilin phosphorylation and actin polymerization. Another reported signaling pathway for latent HIV infection of resting CD4^+^ T cells (dotted line) involves HIV binding to CXCR4; the signal is transferred to CDC42, which activates Wiskott-Aldrich Syndrome family protein (WASP) and actin-nucleating protein (Arp2/3) to promote actin assembly.

In this article, we demonstrate that the chemokine IP-10 plays an important role in the establishment of HIV reservoirs in resting CD4^+^ T cells. We also showed that IP-10 stimulation triggers cofilin activation and actin dynamics in resting CD4^+^ T cells, facilitating latent HIV infection. IP-10 and HIV synergistically increase cofilin and actin activities to promote viral entry and nuclear migration in resting T cells (**Figure 7**). Thus, HIV exploits the early inflammatory response to facilitate the establishment of viral reservoirs in CD4 T cells.

HIV infection can occur in CD4^+^ T cells that have undergone the effector-to-memory transition, which then return to a resting memory state for the establishment of latent reservoirs (31). HIV latent infection can also be established by direct infection of resting memory CD4^+^ T cells (33, 34, 59). However, in the absence of chemotactic signaling, cortical actin in resting CD4^+^ T cells is static and represents a barrier for viral entry and nuclear migration (10, 51, 57). We found that direct infection of resting CD4^+^ T cells by HIV was markedly increased in the presence of IP-10, which promotes actin dynamics necessary for viral entry and nuclear migration.

Cofilin is a key actin regulator essential for T cell migration (10). During HIV latent infection, cofilin is activated by gp120 binding to CXCR4/CCR5 on the surface of resting T cells (10). Cofilin is hyperactivated in resting CD4^+^ T cells in the blood of HIV-infected people, which can lead to impairment in T-cell motility (41, 60, 61). Specifically, cofilin hyperactivation in CCR6^+^ and CXCR3^+^ helper T cells prevented their trafficking from the circulation to peripheral organs such as gut-associated lymphoid tissues (61). Given that these helper T cell subsets harbor the highest amount of integrated HIV DNA (62), their accumulation in peripheral blood may facilitate viral latency and persistence. Cofilin hyperactivation in T cells may be caused by chronic stimulation and chemotactic signaling by HIV gp120; stimulating CD4^+^ T cells *in vitro* with an anti-human α4β7 integrin antibody activated cofilin signaling, partly restoring T cell motility (41). Given the elevated IP-10 levels in HIV-infected individuals, IP-10 may also contribute to cofilin hyperactivation in blood CD4^+^ T cells of HIV-infected individuals. Conversely, reducing IP-10 levels is expected to be beneficial for restoring normal cofilin signaling and alleviating impairment in T cell motility.

In this study, we analyzed a total of 40 chemokines including IP-10 and identified several that were dysregulated in HIV^+^ individuals compared to NCs. Our previous study showed that CXCL9 and CXCL11 levels were significantly increased in primary HIV infection, which was correlated with viral load and AIDS progression (47). Interestingly, CXCL9, IP-10/CXCL10, and CXCL11 have CXCR3 as the common receptor. CXCL9 and CXCL10 are macrophage-related proinflammatory chemokines that are upregulated in the gut mucosa of untreated HIV^+^ individuals, and the levels of these chemokines were shown to be correlated with macrophage accumulation in gut mucosa, which may increase local inflammation and tissue injury (63). Therefore, it is possible that all 3 chemokines promote cofilin activation and latent HIV infection of T cells through CXCR3.

In addition to facilitating the establishment of HIV reservoirs, IP-10 may serve as a biomarker for predicting HIV reservoir size in PBMCs of ART-treated patients; we found that IP-10 level in ART-naïve patients was positively correlated with HIV reservoir size after over 20 months of ART. It was also reported that pre-ART levels of certain chemokines/cytokines such as MCP-1, MIP-3β, soluble TNF receptor-II, and IL-10 are associated with HIV DNA levels after 96 weeks of treatment (64); however, in this particular study, IP-10 level was only correlated with the DNA level in acute infection (Fiebig I/II/III) and not the post-ART level. We analyzed a broader range of time points during primary infection, and our results demonstrate that IP-10 is a stable and reliable biomarker for predicting the size of HIV reservoirs during the whole primary infection phase and not only in Fiebig I/II/III. Moreover, therapeutic targeting of IP-10 is a potential strategy for abolishing latent HIV infection.

Our results showed that IP-10 promotes HIV entry into resting CD4^+^ T cells, and we also found increased viral DNA integration in IP-10-stimulated resting T cells. The increased viral DNA integration likely results from IP-10-induced increases in virus entry, however, we recognize that the increase in viral integration may also result from viral nuclear entry as actin dymatics is also involved in HIV nuclear migration (53).

In this current study, our results suggest that during HIV infection, IP-10 not only promotes inflammation, but also enhances HIV infection of resting memory CD4^+^ T cells. The plasma IP-10 levels before ART may serve as a predictor of HIV viral load and reservoir size after ART. Thus, the use of inhibitors of IP-10 or IP-10-related signal pathways may provide a new strategy for inhibiting the establishment of viral reservoirs.

## Data Availability Statement

The original contributions presented in the study are included in the article/**Supplementary Material**. Further inquiries can be directed to the corresponding authors.

## Ethics Statement

The Research and Ethics Committee of CMU and conducted according to the principles outlined in the Declaration of Helsinki. The patients/participants provided their written informed consent to participate in this study.

## Author Contributions

ZW, XY (2nd author), MM, and HG performed NL4-3/AD8 infection of resting memory CD4+ T cells; gp120 detection; HIV entry and integration, IP-10 signaling, and HIV reservoir experiments. ZW, YJ, YW, and BL wrote the manuscript. ZW, XY (10th author), MM, BL, and YS performed the cytokine/chemokine screen. ZW and HS (6th author) participated in the analysis of HIV reservoirs in HIV^+^ participants. SH and YF participated in the signaling pathway experiments. ZZ, HC, XH collected clinical data of HIV^+^ individuals. HD, ZC, and JX were involved in subject recruitment and evaluation, and collected and analyzed basic information on the subjects. HS (17th author), YW, and YJ conceived, directed, and supervised the study and reviewed the manuscript. All authors contributed to the article and approved the submitted version.

## Funding

This work was funded by research grants from the Mega Projects of National Science Research for the 13th Five-Year Plan (no. 2017ZX10201101), Youth Program of the National Natural Science Foundation of China (no. 81901599), and Mega Projects of National Science Research for the 13th Five-Year Plan (2018ZX10732101-001-011).

## Conflict of Interest

The authors declare that the research was conducted in the absence of any commercial or financial relationships that could be construed as a potential conflict of interest.

## Publisher’s Note

All claims expressed in this article are solely those of the authors and do not necessarily represent those of their affiliated organizations, or those of the publisher, the editors and the reviewers. Any product that may be evaluated in this article, or claim that may be made by its manufacturer, is not guaranteed or endorsed by the publisher.
