# Supplementary material for: IP-10 Promotes Latent HIV Infection in Resting Memory CD4+ T Cells via LIMK-Cofilin Pathway
Source: Front Immunol. 2021 Aug 10;12:656663. doi: 10.3389/fimmu.2021.656663 (PMC8383741; doi:10.3389/fimmu.2021.656663)
Supplement: Supplementary file 1 [file DataSheet_1.doc]

**Supplementary Materials**


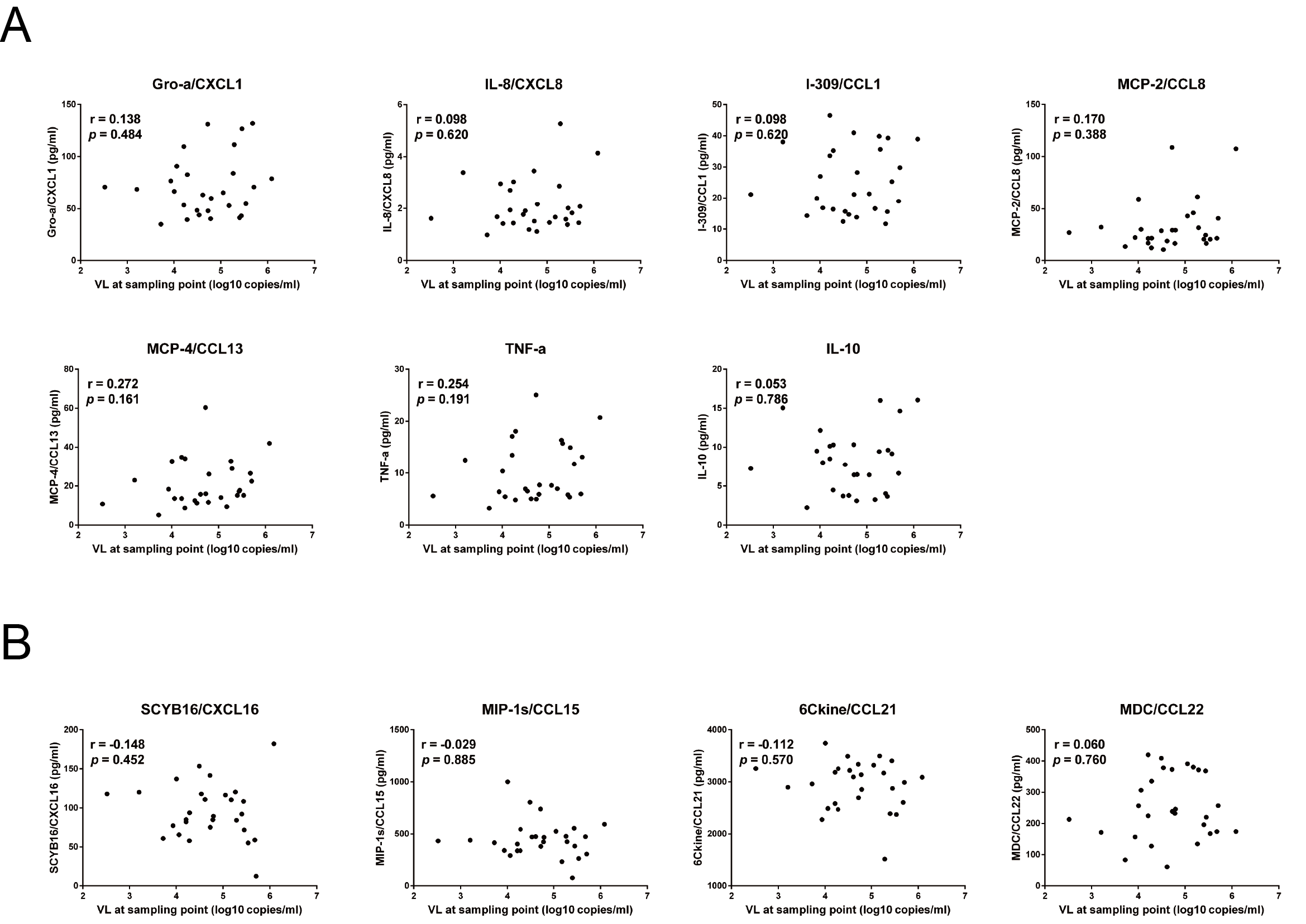


**Figure S1. Alterations in the levels of chemokines and cytokines other than IP-10 show no correlation with HIV viral load.** **(A, B)** Spearman correlation analysis of elevated chemokine (Gro-a/CXCL1, IL-8/CXCL8, I-309/CCL1, MCP-2/CCL8, MCP-4/CCL13, TNF-α, and IL-10) (A) and chemokine (SCYB16/CXCL16, MIP1s/CCL15, 6Ckine/CCL21, MDC/CCL22) (B) levels and viral loads at indicated times.


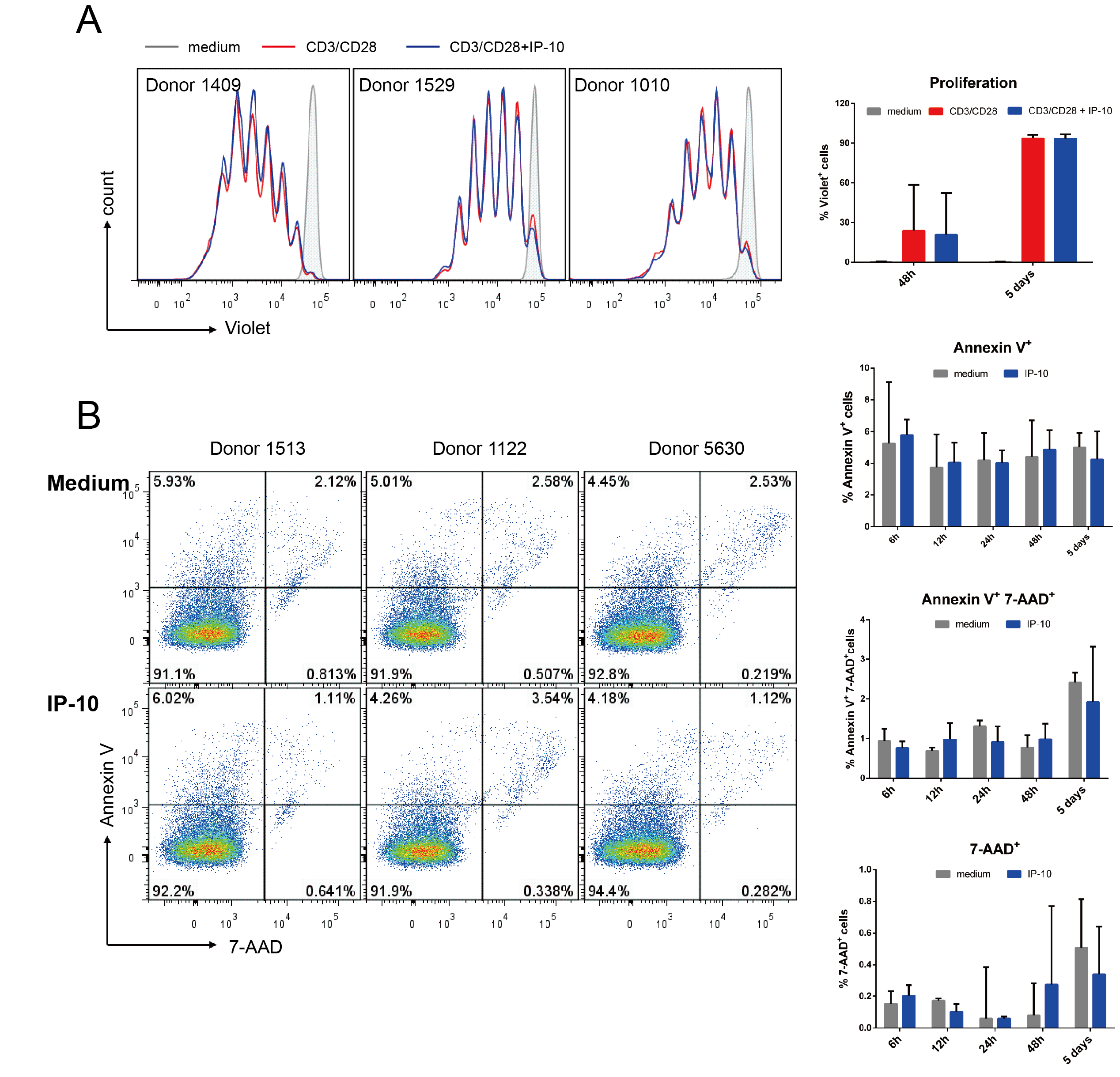


**Figure S2. IP-10 has no effect on the** **proliferation and** **apoptosis levels of resting memory CD4^+^ T cells.** **(A)** Effect of IP-10 on the proliferation of resting memory CD4**^+^** T cells activated by CD3/CD28. Violet dye was used to evaluate the degree of proliferation. **(B)** Effect of IP-10 on apoptosis in resting memory CD4**^+^** T cells evaluated by Annexin V and 7-amino-actinomycin D (7-AAD) staining. Representative flow cytometry plots are shown along with the results of the quantitative analysis. The nonparametric Wilcoxon matched-pairs test was used for intergroup comparisons.

**Table S****1.** Primers and probes used to evaluate integrated HIV DNA level by digital droplet PCR

| Round 1 of PCR | |
| --- | --- |
| L-M667 | ATGCCACGTAAGCGAAACTGGCTAACTAGGGAACCCACTG |
| Alu1 | TCCCAGCTACTGGGGAGGCTGAGG |
| Alu2 | GCCTCCCAAAGTGCTGGGATTACAG |
| Round 2 of PCR | |
| Lambda T | ATGCCACGTAAGCGAAACT |
| AA55M | GCTAGAGATTTTCCACACTGACTAA |
| iVL-P | FAM-AAGCCTCAATAAAGCTTGCCTTGAGTGC-TRAMA |

HIV, human immunodeficiency virus.

**Table S2.** Primers and probes used to evaluate the size of HIV reservoirs

| Primer | LTRG-F | TACTGACGCTCTCGCACC |
| --- | --- | --- |
|  | LTRG-R | TCTCGACGCAGGACTCG |
|  | RPP30-F | GATTTGGACCTGCGAGCG |
|  | RPP30-R | GCGGCTGTCTCCACAAGT |
|  | C1 | CTA ACT AGG GAA CCC ACT GCT |
|  | C4 | GTA GTT CTG CCA ATC AGG GAA G |
| Probe | LTRG | FAM- CTC TCT CCT TCT AGC CTC-MGB |
|  | RPP30 | FAM- CTG ACC TGA AGG CTC T-MGB |
|  | 2-LTR | FAM- AGC CTC AAT AAA GCT TGC-MGB |

HIV, human immunodeficiency virus.

**Table S3.** Characteristics of 29 HIV infected patients for viral reservoir detection

|  | **IP-10 >1000 pg/ml**  **(n=13)** | **IP-10 <1000 pg/ml**  **(n=16)** | ***P* value*** |
| --- | --- | --- | --- |
| IP-10, pg/ml | 2359.66 (1332.06–2872.67) | 445.74 (226.99–586.23) | 0.000 |
| Sex, no. (%) | Male: 11 (85)  Female: 2 (15) | Male: 16 (100) |  |
| Age, years | 43 (30–63) | 37 (27–54) | 0.057 |
| CD4+ T cells, cells/mm³ | 269.00 (222.75–383.75) | 388.50 (285.00–532.25) | 0.026 |
| ART time, months | 30 (20–48) | 28.50 (24.45–48) | 0.820 |
|  |  |  |  |

Data are shown as median (interquartile range) unless otherwise indicated.

*Calculated with the Mann–Whitney U test.

ART, antiretroviral therapy; CD4, cluster of differentiation 4; HIV, human immunodeficiency virus; IP-10, interferon-γ-inducible protein 10; IQR, interquartile range.
